# Tooth-Supported Overdenture: Rediscovering What We Thought We Outgrew

**DOI:** 10.7759/cureus.101708

**Published:** 2026-01-16

**Authors:** Swati Sharma, Rama Shankar, Vijayendra Pandey, Sarat R Kiran, Pankaj Kumar

**Affiliations:** 1 Dentistry, Tata Main Hospital, Jamshedpur, IND; 2 Dentistry, Manipal Tata Medical College, Manipal Academy of Higher Education, Manipal, IND; 3 Oral and Maxillofacial Surgery, Tata Main Hospital, Jamshedpur, IND; 4 Endodontics, Tata Main Hospital, Jamshedpur, IND

**Keywords:** coping overdenture, metal coping, overlay denture, overlay prosthesis, submerged overdenture, tooth-supported overdenture

## Abstract

Edentulism results in impairment, functional limitations, and ultimately psychological and social disabilities. It is a significant factor in residual ridge resorption, leading to a decreased alveolar bone height and a reduced denture-bearing area. A tooth-supported overdenture (TSOD) represents one cost-effective treatment modality for patients requiring removable dentures and helps preserve tooth structure and proprioception, maintain alveolar bone to delay complete tooth loss, provide extra support and stability, and improve chewing efficiency. This case report describes the successful use of a natural TSOD with short metal copings, utilizing the remaining upper root canal-treated teeth as abutments. The lower arch featured a fixed dental prosthesis. Following coping cementation, the overdenture was fabricated using conventional methods, resulting in satisfactory retention, stability, and support. The case is an addendum to the existing literature and demonstrates that it can be performed in a routine setup with minimal additional armamentarium. This also provided the patient with great satisfaction by preventing the loss of all her teeth.

## Introduction

Dental diseases are common in the elderly and often cause tooth loss. Edentulism remains a major global issue, especially in older adults, and negatively impacts oral and overall health. After tooth loss, the dental ridge resorbs, especially in long-term denture wearers, leading to discomfort, poor denture fit, facial changes, and reduced chewing ability [1]. These issues can lower the quality of life and increase requests for new dentures. Edentulous patients are at higher risk for poor nutrition, social and mental health problems, systemic diseases, and increased mortality [1,2]. Bone loss continues after tooth loss, with the mandible more affected than the maxilla. Because of these problems, removing all remaining teeth is not a preferred preventive approach [1,2]. According to Glossary of Prosthodontic Terms-9 (GPT-9), an overdenture is a removable dental prosthesis that covers and rests on one or more remaining natural teeth, the roots of natural teeth, and/or dental implants; a dental prosthesis that covers and is partially supported by natural teeth, natural tooth roots, and/or dental implants [3].

Several prosthetic options are available for patients missing some or all teeth, including removable, fixed, implant-supported, and combined prostheses. Tooth-supported overdentures (TSODs) are reliable but sparingly used. They preserve teeth or roots, reduce bone loss, provide stable support and retention, avoid surgery, maintain proprioception, and offer psychological benefits by allowing patients to retain their own teeth [4,5]. Implant-supported overdentures require less maintenance, allow stronger biting and improved chewing, and can withstand greater occlusal loads, but they are more expensive [5]. Treatment decisions should consider the patient’s health, anatomy, and finances, especially for elderly patients [4,5]. This review examines the benefits and limitations of tooth-supported overdentures, noting that implant-supported overdentures may be preferable in selected cases, but are not always feasible or affordable [4,6]. Reducing the coronal portion of the abutment while maintaining healthy periodontal support improves the crown-to-root ratio and increases abutment longevity. This approach also serves as a transitional solution if the patient later becomes fully edentulous.

Rissin et al. (1978) found that overdenture patients had chewing efficiency one-third higher than those with complete dentures. Various removable and fixed treatments are available for fully edentulous patients. Root-supported overdentures are a proven preventive method, preserving natural teeth or roots and reducing bone loss. They offer better support, improved sensation, and both economic and psychological advantages. Without natural teeth, bone resorbs faster, but retained roots help maintain bone and support the overdenture [7]. Chen et al. (2002) evaluated masticatory efficiency in patients with TSODs compared with those with complete dentures (CDs), concluding that the TSOD provided the highest efficiency, followed by the CD group. As preventive prosthodontics gains prominence, overdentures have emerged as a viable treatment option for patients retaining some natural teeth. The use of natural teeth as overdenture abutments has increasingly replaced extraction as a preferred approach in recent decades [8].

Criteria for abutment and case selection

Proper abutment selection is vital for the success of overdentures. Abutments are placed bilaterally with enough space for roots, copings, attachments, and the denture base. Submerged, non-coping, or coping abutments are chosen as needed. In the short-coping method, teeth are reduced to 1-2 mm above the gingiva, and copings should be at least 4 mm high. Abutments must be restorable, have enough supragingival structure, and have at least 50% bone support. Single-rooted teeth with good canal morphology are preferred. Abutment teeth are assessed for periodontal health and caries, and root canal treatment is performed if needed. Ideally, have one abutment per quadrant, usually canines or premolars, with symmetrical distribution. Non-abutment teeth are extracted. Increased crown-to-root ratio, bone loss, or mobility does not automatically disqualify teeth as abutments, since crown reduction may help. Basal tissue is managed for dentures. A well-adapted base is ensured to distribute forces. The appliance should be easy to fabricate, maintain, and use. Retainers must not hinder insertion or removal to avoid damage. Radiographs are used to check for root stumps, pathology, and periodontal status. This report describes the fabrication of an overdenture that retains maxillary anterior teeth using endodontically treated roots and metal copings to improve outcomes [9-20].

Complications associated with overdentures

Many mandibular denture wearers struggle to adapt due to poor support and stability. Overdentures help preserve ridge height and improve retention. Choosing suitable attachments and a secure interface aids adaptation. Key factors include denture design, border seal, extension, and accurate records. Complications are biological or technical. Caries and periodontal disease mainly cause abutment tooth loss. Regular oral hygiene is critical [9-20]. Older patients may struggle with self-care; for high caries risk, implant-supported prostheses are preferable. Stomatitis, abutment fracture, and apical lesions are other concerns. Technical problems often stem from laboratory errors. Excess gingival margin space leads to dead space and hypertrophy, improved by a displacement wash. Poor attachment fit complicates relining, especially with bar attachments, and may require new dentures. Bone resorption and attachment wear, especially of the matrix, are common and can cause poor fit and fractures. Matrix retention loss is frequent. If root cap retention fails, new dentures or implants are needed. Metal reinforcement reduces fractures. Other issues include coping or attachment failure and root fractures, especially with fewer abutment teeth. More than three abutments may thin the base and increase the risk of fracture. Attachment choice should suit the bone, patient expectations, budget, and expertise. Ongoing maintenance costs should be considered [5,6,9-20]. Tooth-supported overdentures greatly increase patient satisfaction over conventional dentures by improving retention, stability, chewing ability, comfort, and overall quality of life, including confidence and speech. Good oral hygiene prevents plaque buildup, decay on the abutment teeth, gum inflammation, bad breath, and prolongs the life of both the overdenture and the supporting teeth [21-23].

## Case presentation

A 73-year-old female patient presented with an ill-fitting upper complete denture and requested a replacement. The patient's medical history included type II diabetes mellitus, hypothyroidism, and dyslipidemia. The dental history revealed previous upper and lower fixed dental prostheses. She expressed a desire to retain her remaining teeth due to concerns related to diabetes. Clinical examination revealed several anterior maxillary teeth that had undergone root canal treatment. Periodontal status was assessed using a periodontal probe, with depth recorded to 1mm. Mobility was graded according to the Miller classification; class 0 indicates no movement when force is applied. The teeth were resected 3 to 4 mm above the gingival margin. The current overdenture exhibited poor fit, resulting in patient dissatisfaction. The lower arch exhibited a fixed dental prosthesis on all teeth, with both anterior and premolar teeth present bilaterally. A digital intraoral periapical radiograph (IOPAR) of the upper arch confirmed that all remaining teeth were root canal treated and free of infection. Considering the patient's preference to retain her teeth and the limited coronal tooth structure, a tooth-supported overdenture with short metal copings was planned.

**Figure 1:**
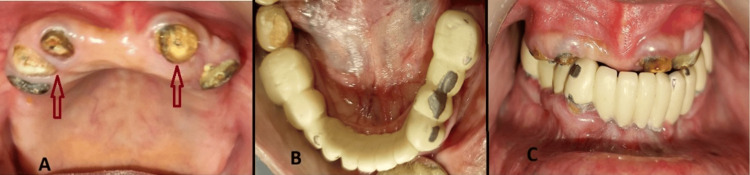
The upper arch displays root canal treatment on the right lateral incisor, canine, and first premolar, as well as the left canine and first premolar (A). The lower arch shows a fixed dental prosthesis (B). Both arches are shown in centric relation (C).

**Figure 2:**
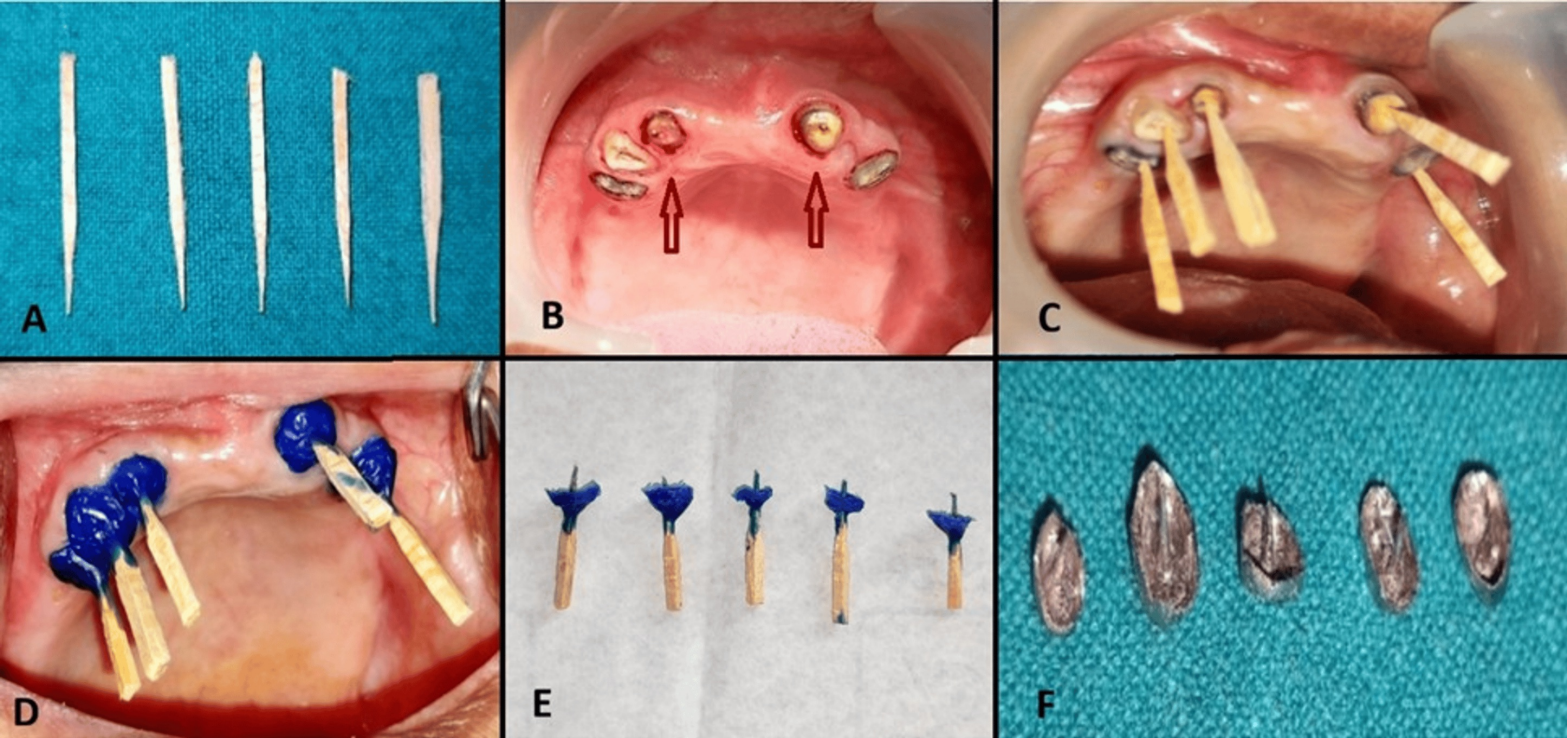
Customised matchsticks (A), teeth preparation with a chamfer finish line for metal copings (B), intra-oral check of customised matchsticks (C), direct wax-up on teeth (D), final wax-up (E), and finished metal copings (F).

The lower fixed dental prosthesis was satisfactory, while the upper bridge had been removed due to caries (Figure 1). Primary impressions were obtained for both arches. Intracanal gutta-percha was removed to enable customization of metal copings with posts. Tooth preparation was completed on all remaining upper teeth using a chamfer finish line. Vaseline was applied as a separating medium. Intraoral wax patterns were fabricated with blue inlay wax and protected to prevent distortion. Following casting, the finished metal copings with posts were evaluated intraorally for fit (Figure 2). 

**Figure 3:**
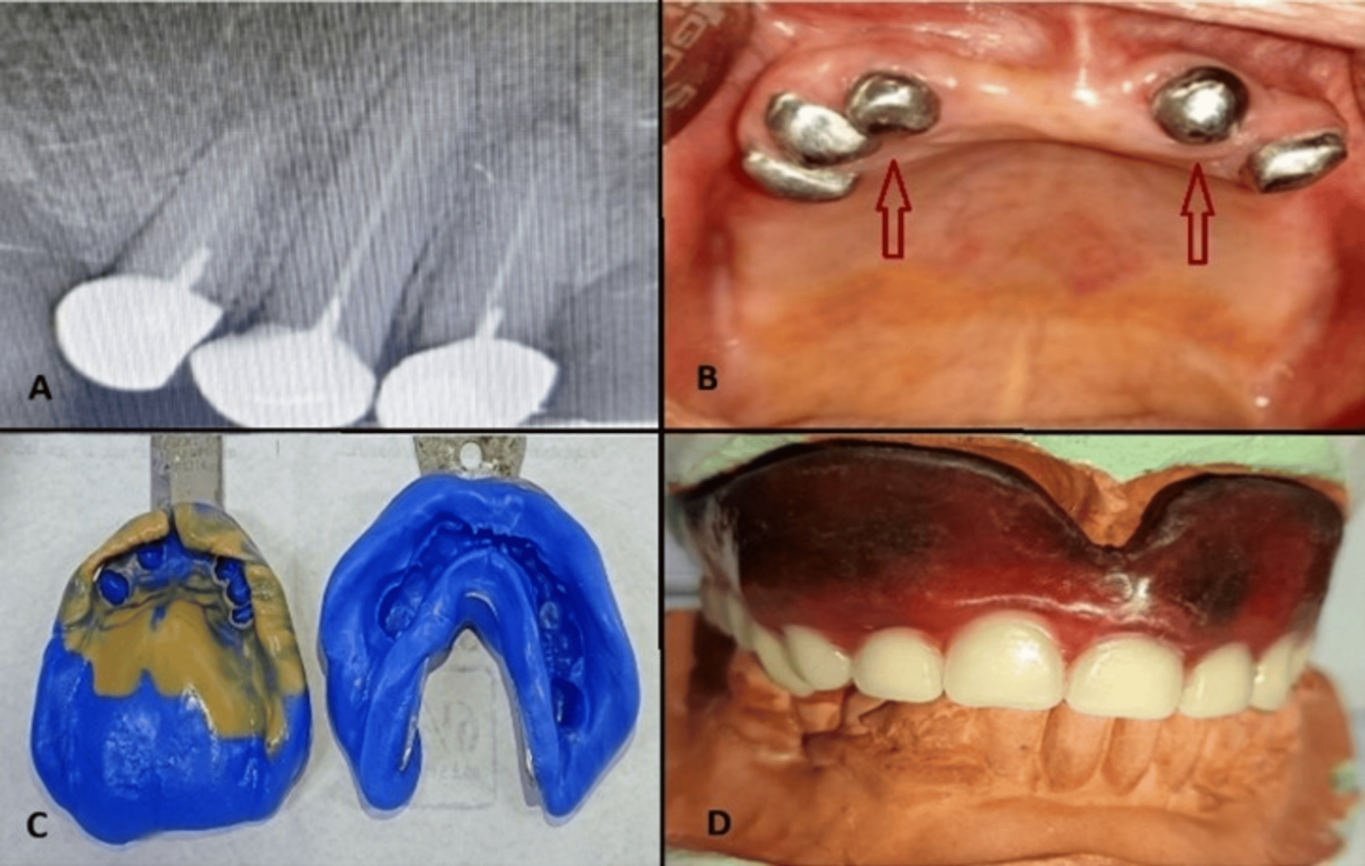
Metal coping in place (IOPAR, A), cementation of metal copings (B), putty impression (C), and try-in (D). IOPAR: Intraoral periapical radiograph.

**Figure 4:**
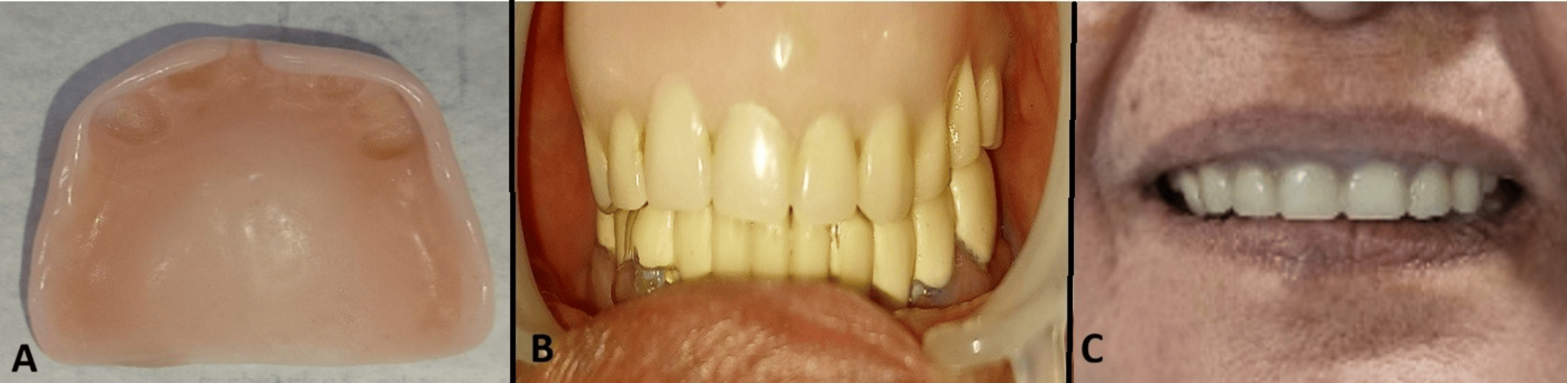
(A) The tissue surface of the upper denture. (B) The intra-oral view in occlusion. (C) The patient, who is satisfied with the outcome.

Once the fit was confirmed, the copings were cemented. A putty impression was subsequently taken, followed by a light-body wash impression after border molding. The maxillomandibular jaw relation was recoded, try-in was done intra-orally (Figure 3), and the final denture was delivered to the patient conventionally (Figure 4). The denture was delivered in September 2024. Follow-up visits revealed no complications or radiographic abnormalities in the abutment teeth. Periodontal status was again recorded at each six‑month follow‑up to allow objective monitoring of abutment health. Both the copings and denture remained in satisfactory condition, and the patient was pleased with the outcome.

## Discussion

A TSOD is recommended for patients with a few remaining natural teeth that are periodontally healthy or have reversible periodontal disease. They are also suitable for individuals with oral conditions like xerostomia, reduced alveolar ridge height, high palatal vault, or unfavorable tongue or muscle positions that may affect prosthesis stability. Poorly positioned or structurally inadequate teeth can be modified to support an overdenture [4-6,9-13]. Overdentures also benefit individuals with rare conditions, such as oligodontia, cleft palate, or anomalies such as dentinogenesis imperfecta and amelogenesis imperfecta [4-6,9-13]. In this case, we selected a TSOD because the patient expressed a desire to retain periodontally healthy, caries-free natural teeth and because no other abnormalities were present.

Although TSOD offers several benefits, certain factors may contraindicate its use. Patient-related barriers include inadequate motivation for oral hygiene, compromised systemic health, financial constraints, high levels of dependency, and restricted access to treatment facilities. Local factors encompass insufficient inter-arch space for denture teeth, especially when additional space is required for abutment restorations or precision attachments. Inadequate space may result in thin acrylic denture bases, thereby increasing the risk of fracture [4-6,9-13]. In this case, none of these contraindications were identified.

Preserving submerged roots or teeth can minimize bone loss, maintain proprioceptive function, improve denture retention, and increase patient satisfaction by preserving natural teeth. Consequently, we supported the patient's preference to retain their natural teeth as they also offered economic and psychological benefits [10-25].

This case involved a complete maxillary TSOD, a configuration less frequently encountered than mandibular TSODs. The prosthesis utilized a tooth-tissue-supported system, distributing occlusal load bilaterally between the abutment teeth and the residual ridge in the posterior regions. Tooth-tissue-supported overdentures are generally more prevalent and demonstrate greater clinical success compared to overdentures supported exclusively by teeth. In this instance, the presence of three or more abutment teeth enhanced both retention and stability of the prosthesis [9-20].

Overdentures can be classified according to various variables described in Table 1 [9-20].

**Table 1:** Classification of overdentures The table is manually created by the authors after going through the relevant literature [9-20].

Classification Criteria	Types/Classes	Description/ Key Points
Classification Based on the Timing of Insertion	Immediate overdenture	Inserted immediately after tooth extraction, using modified natural teeth or implants for support; serve as a long- term prosthesis if conditions allow.
	Transitional overdenture	Temporary prosthesis converted from an existing partial denture during the healing period post-extraction; bridges to a definitive prosthesis.
	Remote/Definitive overdenture	Constructed and inserted well after extraction and healing, typically as a permanent solution.
Based on Attachment Type	Precision attachment	Custom-made (e.g., Hader bar, Dolder bar, Ceka, Rothermann)
	Semi-precision attachment	Pre-fabricated plastic patterns cast in metal
	Resilient/Non-precision attachment	O-ring, Locator, Ball, Extracoronal Resilient Attachment (ERA), Magnet
Based on Support	Entirely tooth-supported	All load on abutment teeth (very rare)
	Tooth-tissue supported (most common)	Load shared between teeth and the residual ridge
Based on the Number of Abutments	Single abutment overdenture	Rare, usually midline
	Two abutment overdenture	Most common (usually bilateral canines)
	Three or more abutment overdentures	Increased stability
Andrews’ Classification (1970s)	Class I: Bilateral support (canine/premolar)	Most favorable prognosis
	Class II: Unilateral support	-
	Class III: Unilateral with midline separation	-
	Class IV: Anterior support only	Least favorable
Based on Coping Design	No coping (only RCT tooth)	-
	Short coping (dome-shaped)	Most common
	Long coping ± post	-
	Bar attachment (splinted)	Connects multiple abutments
Based on the Retention Mechanism	Stud attachments	Locator, Ball, O-ring, ERA, Dalla Bona (most popular)
	Bar attachments	Dolder (egg-shaped), Hader (U-shaped), custom milled
	Magnet attachments	-
	Telescopic crowns (double crown system)	Conical or parallel-walled copings
Based on Jaw	Maxillary tooth-supported overdenture	Less common
	Mandibular tooth-supported overdenture	Far more common & successful (prevents combination syndrome)

A fixed dental prosthesis was present in the lower arch and was retained due to its asymptomatic status and satisfactory function. In the upper arch, five root canal-treated teeth were identified: the lateral incisors, canine, and first premolar in the first quadrant, as well as the canine and first premolar in the second quadrant. These single-rooted teeth exhibited favorable canal morphology and were deemed suitable for metal copings, with adequate supragingival structure. The abutment teeth showed at least 50% bone support and minimal mobility, as confirmed by IOPAR [17-20].

The abutment teeth exhibited a remaining height of 3-4 mm; therefore, a short metal coping design, which is the most common approach, was selected. Radiographs were obtained to exclude root stumps, detect pathology, and evaluate periodontal status. In the short-coping method, teeth are reduced to 1-2 mm above the gingiva, and copings should be at least 4 mm high to minimize fracture risk, as was implemented in this case. Although extraction of non-abutment teeth is generally recommended, it was not required in this instance [17-29].

The cast coping fabrication may increase costs; this was not a concern for the patient. A single-step primary and secondary impression was obtained using putty and light body materials. Maxillo-mandibular jaw relations were recorded, followed by a try-in, and the denture was fabricated using conventional techniques. The lack of significant bony undercuts adjacent to the abutment teeth permitted full extension of the denture flange without compromising retention or aesthetics. Basal tissue management was effective, resulting in a well-adapted base that facilitated even distribution of occlusal forces. The denture demonstrated ease of fabrication, maintenance, and use. Additionally, the metal coping housing did not impede insertion or removal, thereby minimizing the risk of damage to the base or abutments [17-29].

One notable limitation of overdentures is the difficulty in maintaining optimal oral hygiene. Regular recall appointments were scheduled to reinforce hygiene practices and to apply topical fluoride, thereby reducing the risk of caries and periodontal disease in abutment teeth. Radiographs were obtained at intervals of no more than six months to monitor dental health. Following denture insertion, the patient received comprehensive oral and denture hygiene instructions and adhered to them, thereby preventing hygiene-related complications [4-20].

After denture delivery to the patient, no complications or radiographic abnormalities were observed in the abutment teeth during subsequent follow-up visits. Both the copings and the denture remained in satisfactory condition. The visual analogue scale score for mastication showed a score of zero [30]. 

## Conclusions

Overdentures offer certain benefits over regular full dentures by maintaining proprioception and delaying bone resorption. How well they work depends on choosing the right cases and carefully managing treatment. Replacing missing teeth with overdentures helps people chew and look better, and can also boost confidence, especially for younger patients. Dentists should consider TSODs before extracting all remaining teeth, assessing whether the existing teeth can support overdentures. There are many types of TSODs, with different prices and levels of difficulty, so dentists can pick what works best for each person. The case is an addendum to the existing literature and has shown that it can be done in a routine setup with minimal additional armamentarium. Further studies are recommended to support the outcome on a larger scale. 
